# Medical Chart

**Published:** 1875-06

**Authors:** 


Medical Chart.—We have received specimens of the Medical Chart, published by the Case Record Company, of Cincinnati. It is designed for the register of cases at the bedside, and is, we think, well adapted for the purpose in view. The prices charged, five cents each, fifty cents per dozen, three dollars per hundred, will likely prove an obstacle to their general introduction.

---

We learn that Dr. E. J. Kirkscey, of Columbus, Ga., for sometime resident at Louisville, Ky., has been honored with the offer of a surgeon's commission in the army of the Khedive, which he accepts, and will leave for Egypt in July next. We wish him good health and a long and useful life with his newly chosen friends in the orient.

